# SCMTHP: A New Approach for Identifying and Characterizing of Tumor-Homing Peptides Using Estimated Propensity Scores of Amino Acids

**DOI:** 10.3390/pharmaceutics14010122

**Published:** 2022-01-04

**Authors:** Phasit Charoenkwan, Wararat Chiangjong, Chanin Nantasenamat, Mohammad Ali Moni, Pietro Lio’, Balachandran Manavalan, Watshara Shoombuatong

**Affiliations:** 1 Modern Management and Information Technology, College of Arts, Media and Technology, Chiang Mai University, Chiang Mai 50200, Thailand; phasit.c@cmu.ac.th; 2 Pediatric Translational Research Unit, Department of Pediatrics, Faculty of Medicine, Ramathibodi Hospital, Mahidol University, Bangkok 10400, Thailand; wararat_01@yahoo.com; 3 Center of Data Mining and Biomedical Informatics, Faculty of Medical Technology, Mahidol University, Bangkok 10700, Thailand; chanin.nan@mahidol.edu; 4 Artificial Intelligence & Digital Health Data Science, School of Health and Rehabilitation Sciences, Faculty of Health and Behavioural Sciences, The University of Queensland, St Lucia, QLD 4072, Australia; m.moni@uq.edu.au; 5 Department of Computer Science and Technology, University of Cambridge, Cambridge CB3 0FD, UK; pl219@cam.ac.uk; 6 Department of Physiology, Ajou University School of Medicine, Suwon 16499, Korea

**Keywords:** tumor-homing peptide, therapeutic peptide, scoring card method, propensity score, machine learning, bioinformatics

## Abstract

Tumor-homing peptides (THPs) are small peptides that can recognize and bind cancer cells specifically. To gain a better understanding of THPs’ functional mechanisms, the accurate identification and characterization of THPs is required. Although some computational methods for in silico THP identification have been proposed, a major drawback is their lack of model interpretability. In this study, we propose a new, simple and easily interpretable computational approach (called SCMTHP) for identifying and analyzing tumor-homing activities of peptides via the use of a scoring card method (SCM). To improve the predictability and interpretability of our predictor, we generated propensity scores of 20 amino acids as THPs. Finally, informative physicochemical properties were used for providing insights on characteristics giving rise to the bioactivity of THPs via the use of SCMTHP-derived propensity scores. Benchmarking experiments from independent test indicated that SCMTHP could achieve comparable performance to state-of-the-art method with accuracies of 0.827 and 0.798, respectively, when evaluated on two benchmark datasets consisting of Main and Small datasets. Furthermore, SCMTHP was found to outperform several well-known machine learning-based classifiers (e.g., decision tree, k-nearest neighbor, multi-layer perceptron, naive Bayes and partial least squares regression) as indicated by both 10-fold cross-validation and independent tests. Finally, the SCMTHP web server was established and made freely available online. SCMTHP is expected to be a useful tool for rapid and accurate identification of THPs and for providing better understanding on THP biophysical and biochemical properties.

## 1. Introduction

Tumor-homing peptides (THPs) are short peptides ranging in size from 3 to 30 residues that specifically target tumor cells [1]. THPs may be used in the near future for tumor diagnostic and therapeutic applications due to their low antigenicity, lack of significant cytotoxicity to normal cells, rapid incorporation into target cells as well as their ease of modification and redesign [2]. THPs’ motifs frequently contain RGD (Arg-Gly-Asp) and NGR (Asn-Gly-Arg), which are known to favor receptor-mediated interaction in cancer cell recognition with high specificity and low cross-reactivity [3,4]. Furthermore, the RGD function is compatible with KGD [5], RYD [6] and RHDS [7] motifs in integrin binding sites [8]. Hundreds of THPs could identify tumors in vivo and deliver anticancer drugs to the tumor site, thereby resulting in cancer treatment and diagnosis [9]. THPs were developed as a targeted vehicle for gene therapy of solid tumors such as the sodium-iodide symporter (NIS) [10]. So far, several THPs have been experimentally tested in clinical trials. For example, in a phase I/II trial testing for safety and immunogenicity, a multipeptide vaccine (IMA950) formulated the nine antigens by peptide elution from the surface of glioblastoma [11,12]. THPs’ experimental characterization, on the other hand, is still a time-consuming and labor-intensive endeavor. As a result, approaches based on machine learning (ML) that can accurately identify THPs based on primary sequence information would be beneficial. Furthermore, these methods may reveal important information about THPs’ functional mechanisms.

To the best of our knowledge, only two computational methods in the field have been made (TumorHPD [13] and THPep [14]). These two approaches have been developed to identify THPs solely based on their sequence information (e.g., amino acid composition (AAC) and dipeptide composition (DPC)). Sharma et al. [13] proposed the first THP predictor (TumorHPD) in 2013, which was created using a support vector machine (SVM) algorithm in conjunction with AAC, DPC and binary profile patterns (BPP). Furthermore, in this research work Sharma et al. shared two benchmark datasets namely Main and Small datasets. Our group proposed the second THP predictor (referred to as the THPep [14]) by combining the use of the random forest (RF) algorithm with three popular sequence-based feature descriptors (e.g., AAC, DPC and pseudo amino acid composition (PAAC)). THPep was found to improve the overall performance in terms of accuracy (ACC), sensitivity (Sn), Matthew’s Correlation Coefficient (MCC) and area under the receiver-operating curves (AUC) when compared to TumorHPD (i.e., as measured by the cross-validation test on the two benchmark datasets) [13]. Although the performance of these two existing THP predictors was generally good, there was a strong need for new approaches that can yield good prediction performance while also providing biologists mechanistic interpretation of tumor homing activities of peptides that can be used for guiding the design of robust peptides.

**Figure 1:**
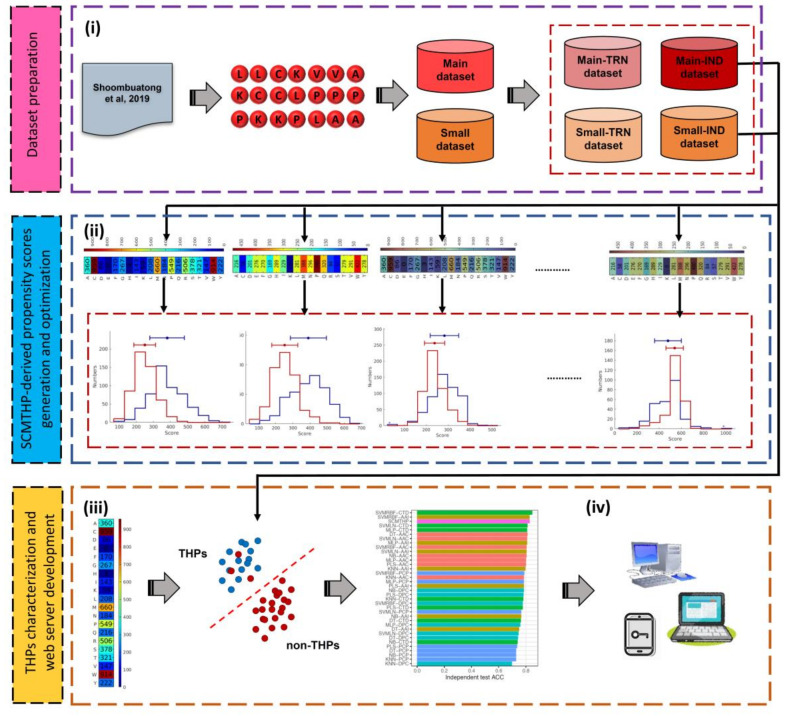
Schematic framework of the development of SCMTHP. This can be broken down to four major steps: (**i**) training and independent datasets preparation, (**ii**) SCMTHP-based propensity scores generation and optimization, (**iii**) THPs characterization and (**iv**) SCMTHP webserver construction.

To address the aforementioned issues, we have developed SCMTHP as a novel, simple and interpretable method for in silico identification and characterization of peptide tumor homing activities using primary sequence information. Figure 1 summarizes the SCMTHP schematic framework for THP identification and characterization. Particularly, the major contributions of SCMTHP can be summarized as follows.

1. To the best of our knowledge, SCMTHP is the first propensity score-based predictor that is employed to create and optimize several new propensity scores of 20 amino acids in becoming THPs via the scoring card method (SCM) [15,16,17]. In the meantime, a single feature descriptor (i.e., AAC) and a single threshold value were implemented in the SCMTHP predictor, and it was found that the approach could easily distinguish THPs from non-THPs.
2. Extensive benchmarking experiments show that SCMTHP could outperforms almost all ML-based predictors (e.g., decision tree (DT), k-nearest neighbor (KNN), multi-layer perceptron (MLP), naive Bayes (NB) and partial least squares regression (PLS)) as well as state-of-the-art THP predictors in terms of accuracy, cost-effectiveness and simplicity.
3. In order to characterize tumor-homing activities of peptides, SCMTHP-derived propensity scores of 20 amino acids were employed to determine informative physicochemical properties (PCPs) of amino acids as provided in the AAIndex database [18]. The importance of Cys residue in stabilization and the preference for high extinction coefficients are revealed by an analysis of SCMTHP-derived propensity scores.
4. A user-friendly online web server was built and deployed publicly at http://pmlabstack.pythonanywhere.com/SCMTHP (accessed on 27 December 2021) in order to facilitate online high-throughput THP identification. We believe that the SCMTHP predictor and SCMTHP-derived propensity scores will be helpful in facilitating THP identification as well as improving our understanding of their biophysical and biochemical properties.

## 2. Materials and Methods

### 2.1. Dataset Preparation

In order to conduct a fair test, the proposed method was optimized and evaluated using the same benchmark datasets (i.e., the Main and Small datasets) as performed in our previous work [14]. Sharma et al. [13] originally compiled these two benchmark datasets. Particularly, there are 1302 sequences in the Main dataset (651 THPs and 651 non-THPs) and 938 sequences in the Small dataset (469 THPs and 469 non-THPs). The THP samples in the Main dataset were obtained from the TumorHoPe [19] database and were experimentally validated THPs whereas non-THP samples were obtained by randomly selecting peptides from SwissProt [13]. Particularly, the Small dataset was created by taking a subset of the Main dataset and selecting peptides in the range of 4 and 10 residues. To test the model’s effectiveness, 161 THPs and 161 non-THPs were chosen at random from the set of 1302 sequences that will be referred to as the independent dataset (Main-IND) while the remaining sequences were used as the training dataset (Main-TRN) (490 THPs and 490 non-THPs). Similarly, 119 THPs and 119 non-THPs were chosen at random from the set of 938 sequences to form the independent dataset (Small-IND) while the remaining sequences formed the training dataset (Small-TRN) (350 THPs and 350 non-THPs). Note that the two benchmark datasets along with their training and independent datasets can be downloaded from http://pmlabstack.pythonanywhere.com/dataset_SCMTHP (accessed on 27 December 2021).

### 2.2. Scoring Card Method

SCM has been shown to afford good predictive performance as well as achieve comparable results to those of popular ML classifiers [20,21,22,23]. The contribution of the SCM method is summarized in the following three aspects. First, unlike complex methods such as SVM and RF, the SCM method can discriminate positive samples from negative samples using only the simple weighted-sum function. This emphasizes its ease-of-use and interpretability [24,25]. Second, as the SCM method is based on a single feature descriptor (i.e., AAC or DPC) and a threshold value, which suggests that this method could achieve better computational efficiency as compared to other conventional complex methods [26,27]. Third, the estimated propensity scores of 20 amino acids and 400 dipeptides enables an automatic identification of informative PCPs provided in the AAIndex database [18] that might be useful for characterizing and analyzing various functions of proteins and peptides. Below is a detailed description on the estimation of SCM-derived propensity scores and construction of the SCMTHP model using the Main dataset.

Phase 1: The training (Main-TRN) and independent (Main-IND) datasets are prepared. Particularly, the Main-TRN dataset was employed to determine the optimized propensity scores of 20 amino acids (Optimized-APS). Afterwards, the Optimized-APS was used to estimate the threshold value for the identification of unknown peptides as THPs or non-THPs.

Phase 2: Computing the ratio between each amino acid by the occurrence frequency of aa(i) for THP and non-THP classes to generate the initial propensity scores of 20 amino acids (Initial-APS). Taking Cys as an example, the frequency of Cys in THP and non-THP classes was 650 and 200, respectively. The normalized Cys compositions in THP and non-THP classes were 0.6 and 0.2, respectively. Finally, we normalized the score of each amino acid to be in the range of 0–1000 in order to facilitate the feature analysis. Our previous studies provide more information on how Initial-APS are calculated [15,17].

Phase 3: Genetic algorithm (GA) was used for optimizing the Initial-APS in order to maximize the predictive performance and to preserve the original information of THPs [17,28]. Particularly, the GA’s fitness function ($\mathrm{Fit}\left(\mathrm{APS}\right)$) was defined by the area under the receiver-operating curve (ROC) curve (AUC) value and the Pearson’s correlation coefficient (R value) between the Initial-APS and Optimized-APS.

(1) $$\mathrm{Fit}\left(\mathrm{APS}\right)=W_{1}\times \mathrm{AUC}+{\;W}_{2}\times R$$

where $W_{1}=0.9$ and $W_{2}=0.1$. Weights for $W_{1}$ and $W_{2}$ were directly obtained from our previous studies [16,17]. Note that the $\mathrm{Fit}\left(\mathrm{APS}\right)$ function was performed using a 10-fold cross-validation procedure in order to avoid the overfitting issue. A detailed description on the determination of Optimized-APS by means of the GA algorithm is provided in the Supplementary information.

Phase 4: Constructing a scoring function $\mathrm{SF}\left(P\right)$ based on the Optimized-APS. The $\mathrm{SF}\left(P\right)$ function was used to calculate THP scores for query peptides P. The $\mathrm{SF}\left(P\right)$ function can be defined as follows:

(2) $$\mathrm{SF}\left(P\right)=\overset{20}{\underset{i=1}{\sum }}{\mathrm{aa}}_{i}{\mathrm{APS}}_{i}$$

where $aa_{i}$ and $APS_{i}$ represent the occurrence frequency and propensity score of the ith amino acid.

Phase 5: Identifying the biological function of a query peptide P and determining the optimal threshold value (Cutoff) yielding the highest cross-validation performance. For a given unknown peptide P, it is classified as THP if $\mathrm{SF}\left(P\right)$ is greater than the Cutoff otherwise P is classified as non-THP.

(3) $$\mathrm{Pred}\left(P\right)=\left\{\begin{array}{c} 1,\overset{20}{\underset{i=1}{\sum }}{\mathrm{aa}}_{i}{\mathrm{APS}}_{i}>\mathrm{Cutoff}\\ 0,\overset{20}{\underset{i=1}{\sum }}{\mathrm{aa}}_{i}{\mathrm{APS}}_{i}<\mathrm{Cutoff} \end{array}\right.$$

where 1 and 0 represent THP and non-THP classes, respectively. For the Small dataset, its propensity scores can be calculated in the same process without significant modifications.

### 2.3. Characterization of THPs Using Informative Physicochemical Properties

To characterize the tumor-homing activities of peptides, the propensity scores of 20 amino acids were used to identify the important PCPs from the AAindex database [18]. The following steps were used to determine the set of informative PCPs using SCMTHP: (i) PCPs with the value ‘NA’ were not included in this study. As for the remaining 531 PCPs, we computed R values for propensity scores of 20 amino acids and each of the 531 PCPs, and (ii) if the R values were >0.5, these PCPs were considered as candidate PCPs for THPs analysis. Note that PCPs with the highest R values were deemed to be the most important.

### 2.4. Conventional ML-Based Classifiers 

SCMTHP was compared to ML-based classifiers trained with various ML algorithms (DT, KNN, MLP, NB, PLS and SVM) and sequence-based feature descriptors (AAC, DPC, PCP, amino acid index (AAI) and composition-transition-distribution (CTD)). In addition, linear (namely, SVMLN) and radial basis function (namely, SVMRBF) kernels were utilized for constructing SVM-based classifiers. The five different sequence-based feature descriptors were extracted using the iFeature module in the Python environment [20]. The Scikit-learn package in Python (version 0.22) was then used to generate ML classifiers for each feature descriptor individually [29]. The optimal parameters of MLP-based, SVMLN-based and SVMRBF-based classifiers were determined using a 10-fold cross-validation procedure on the training (Main-TRN and Small-TRN) datasets, where the search range is shown in Supplementary Table S1. In the meantime, the remaining ML-based classifiers were implemented with their default parameters. Using the Scikit-learn package in Python (version 0.22) [29], 35 ML-based classifiers (7 MLs × 5 descriptors) were created in this study.

### 2.5. Performance Evaluation

Five common performance measures consisting of ACC, Sn, MCC, AUC and specificity (Sp) [30,31] were used to evaluate the predictive performance of our proposed model, the compared ML-based THP classifiers and the state-of-the-art method. These performance measures are defined as follows:

(4) $$\mathrm{ACC}=\frac{\mathrm{TP}+\mathrm{TN}}{\left(\mathrm{TP}+\mathrm{TN}+\mathrm{FP}+\mathrm{FN}\right)}$$

(5) $$\mathrm{Sn}=\frac{\mathrm{TP}}{\left(\mathrm{TP}+\mathrm{FN}\right)}$$

(6) $$\mathrm{Sp}=\frac{\mathrm{TN}}{\left(\mathrm{TN}+\mathrm{FP}\right)}$$

(7) $$\mathrm{MCC}=\frac{\mathrm{TP}\times \mathrm{TN}-\mathrm{FP}\times \mathrm{FN}}{\sqrt{\left(\mathrm{TP}+\mathrm{FP}\right)\left(\mathrm{TP}+\mathrm{FN}\right)\left(\mathrm{TN}+\mathrm{FP}\right)\left(\mathrm{TN}+\mathrm{FN}\right)}}$$

where TP, TN, FP and FN represent the number of true positives, true negatives, false positive and false negatives, respectively [32,33,34].

## 3. Results and Discussion

### 3.1. Performance of Different Propensity Scores

In this section, we used 10-fold cross-validation and independent tests to investigate and evaluate the performance of variant SCM models trained using different sets of Optimized-APS on the two benchmark datasets (i.e., the Main and Small datasets). In this study, ten independent runs were performed for each of the two benchmark datasets to generate ten different sets of Optimized-APS using the GA algorithm, which were then used to construct ten different SCM models. Supplementary Tables S2–S5 summarize the cross-validation and independent test results, respectively.

**Figure 2:**
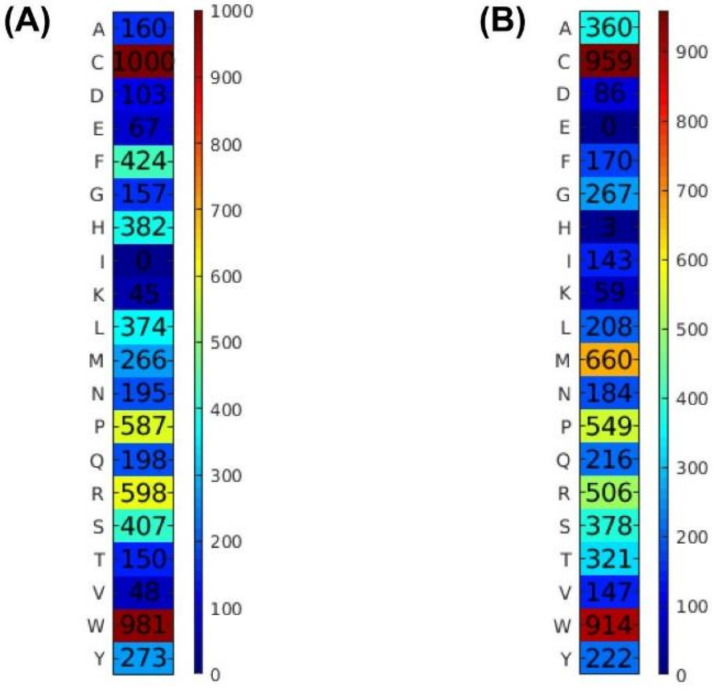
Propensity scores of 20 amino acids to be THPs by using the SCMTHP method with the Main (**A**) and Small (**B**) datasets.

As can be seen from the Supplementary Table S2, the 10th experiment achieved the highest ACC of 0.820 with an MCC of 0.641 and an AUC of 0.869. Furthermore, the 7th and 2nd experiments achieved the second and third highest prediction results, respectively. Interestingly, the 10th experiment also provided the best independent test result in terms of all performance metrics on the Main-IND dataset. To be specific, the ACC, MCC and AUC from the 10th experiment had values of 0.827, 0.656 and 0.869, respectively (Supplementary Table S3). In case of the Small dataset, the 3rd and 7th experiments were found to achieve superior performance when compared with other experiments as evaluated on the Small-TRN dataset (Supplementary Table S4). Notably, the 3rd experiment could achieve the best independent test result as indicated by three out of five performance metrics (ACC, Sp and MCC) on the Small-IND dataset. Particularly, the ACC, Sp and MCC from the 3rd experiment had corresponding values of 0.798, 0.830 and 0.597, respectively (Supplementary Table S5). Altogether, SCM models were constructed using the Optimized-APS from the 10th and 3rd experiments (Figure 2), respectively, for the Main and Small datasets that is referred herein as SCMTHP. In addition, these two sets of Optimized-APS will be employed for further analysis.

### 3.2. Comparison of SCMTHP with Well-Known ML Classifiers and Existing Methods

**Figure 3:**
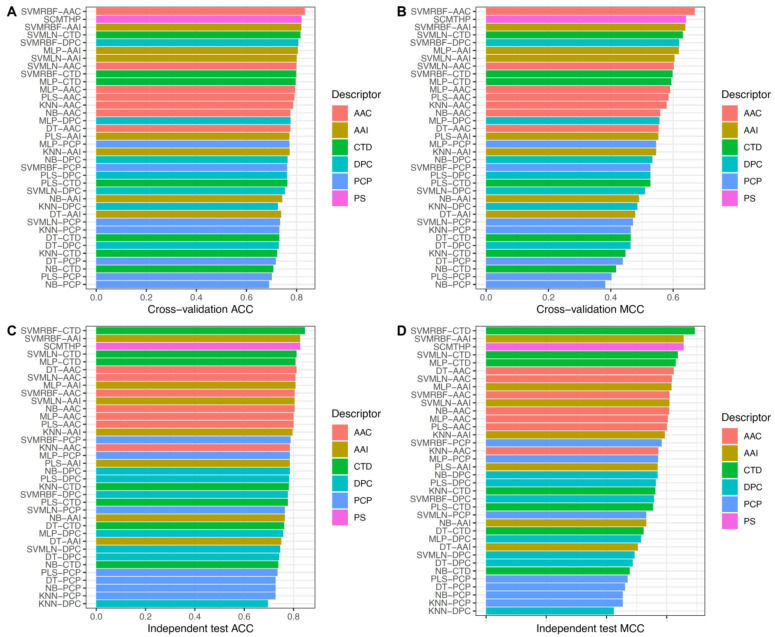
Performance evaluations of SCMTHP and other ML-based classifiers in terms of ACC and MCC as evaluated by 10-fold cross-validation (**A**,**B**) and independent (**C**,**D**) tests on the Main-TRN and Main-IND datasets, respectively.

In this section, we compared the predictive performance of SCMTHP with conventional ML classifiers as well as state-of-the-art method. To ensure fairness and objectivity, all of the compared ML-based classifiers and state-of-the-art method were developed and evaluated using the same training (i.e., the Main-TRN and Small-TRN) and independent (i.e., the Main-IND and Small-IND) datasets as presented in THPep [14]. Particularly, there are two existing methods that had been developed for THP identification (TumorHPD [13] and THPep [14]). However, THPep is the only existing method that was developed and evaluated based on the above-mentioned benchmark datasets. Therefore, the performance of SCMTHP was compared with THPep only. Results from comparing SCMTHP with conventional ML classifiers and state-of-the-art method are shown in Figure 3, Table 1 as well as Supplementary Figure S1 and Tables S6–S9.

Figure 3 and Supplementary Figure S1 revealed several observations as follows. First, among the seven ML-based classifiers that are compared, the SVMRBF-based classifier was found to achieve the best mean ACC of 0.804 and 0.775 as evaluated on the Main-TRN and Small-TRN datasets, respectively, while SVMLN-based (0.780, 0.763) and MLP-based (0.788, 0.756) classifiers achieved a similar level of performance and could perform well with the second highest performance. Second, it could be observed that SVMRBF-AAC and SCMTHP could achieve the best cross-validation performance (ACC, MCC) of (0.833, 0.669) and (0.808, 0.628) on the Main-TRN and Small-TRN datasets, respectively. For the Main-TRN dataset, SCMTHP produced ACC and MCC of 0.820 and 0.641, respectively, which was very comparable to that of SVMRBF-AAC. Third, the SCM-based classifier was found to outperform DT-based, KNN-based, MLP-based, NB-based, PLS-based and SVMLN-based classifiers on both the Main-TRN and Small-TRN datasets. Fourth, independent test results indicated similar results to that observed from the cross-validation test. Particularly, SCMTHP achieved ACC of 0.827 and 0.798 on Main-IND and Small-IND datasets, respectively, which outperformed several ML-based classifiers as developed in this study with the exception of SVMRBF-CTD.

We also put the SCMTHP to the test and compared it to THPep. The ACC of SCMTHP as evaluated on Main-IND and Small-IND datasets provided corresponding values of 0.827 and 0.798, respectively, which were comparable to that of THPred (0.846 and 0.798, respectively) (Table 1). It was recognized that THPred was created by combining the complex ensemble method (i.e., the RF algorithm) with AAC and PAAC [14]. On the other hand, SCMTHP was trained using a simple weighted-sum classifier ($\mathrm{SF}\left(P\right)$) and a single feature descriptor (i.e., AAC). Such model could provide us with the propensity scores of 20 amino acids to be THPs in an easily interpretable manner from a biologist’s perspective. In terms of accuracy, cost-effectiveness and simplicity, the proposed SCMTHP could outperform the compared ML-based classifiers and the state-of-the-art method.

### 3.3. Contribution of Optimized Propensity Scores 

**Figure 4:**
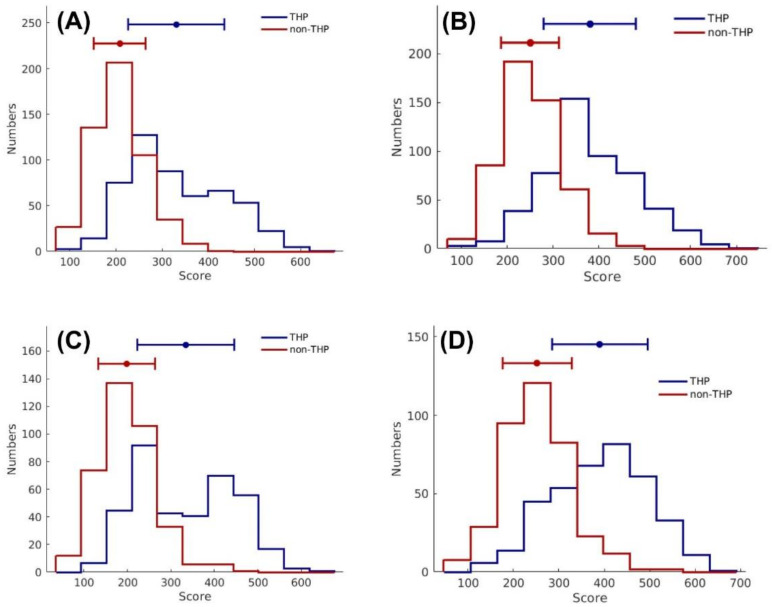
Histogram plots of THPs’ scores and non-THPs’ scores from SCMTHP on the Main-TRN (**A**,**B**) and Small-TRN (**C**,**D**) datasets by using Initial-APS (**A**,**C**) and Optimized-APS (**B**,**D**). Note that the mean and standard deviation are indicated by the bars and closed circles.

As previously stated, the SCM approach was used to generate and optimize propensity scores of 20 amino acids in governing its contribution of becoming THPs in order to maximize their predictive ability and interpretability. The performance of the Optimized-APS was compared to the Initial-APS using 10-fold cross-validation and independent tests on the Main and Small datasets. Supplementary Table S10 shows the detailed performance of the Optimized-APS and the Initial-APS. Note that the Optimized-APS demonstrated the best overall predictive performance across the board in terms of all five performance metrics. On the Main-TRN and Main-IND datasets, the Optimized-APS had maximum cross-validation and independent test MCC of 0.641 and 0.626, respectively, which are correspondingly 13% and 17.6% higher than the Initial-APS (0.511 and 0.480, respectively). Interestingly, the Optimized-APS could outperform the Initial-APS in four out of five performance metrics as evaluated on the Small-TRN and Small-IND datasets (i.e., ACC, Sp, MCC and AUC). Remarkably, the ACC, Sp, MCC and AUC for Optimized-APS had values of 6.9%, 17.0%, 13.5% and 1.3%, respectively, which was higher than that of the Initial-APS. Moreover, as can be seen from Figure 4, Optimized-APS exhibited more discriminative ability in classifying THPs from non-THPs than that of Initial-APS as evaluated on the Main (Figure 4A,B) and Small datasets (Figure 4C,D). The aforementioned results confirmed that the proposed Optimized-APS (i.e., propensity scores of 20 amino acids or SCMTHP-derived propensity scores) was effective at discriminating THPs from non-THPs.

### 3.4. Identification of Potential THPs Using SCMTHP-Derived Propensity Scores

This section explores the use of SCMTHP for measuring the tumor homing ability of peptides using THP score calculated from a simple weighted-sum function (S(P)). This weighted-sum function was generated using the propensity scores of 20 amino acids from the 10th experiment where the threshold value is set to 301 (Supplementary Table S2). It should be noted that peptide sequences with the highest THP scores could be considered as high-potential THPs. As can be seen from Table 2 and Table 3, several observations can be summarized as follows. First, mean, maximum and minimum THP scores for the top 20 THPs had corresponding values of 610.25, 684 and 571, respectively, while the mean, maximum and minimum scores of the top 20 non-THPs were 149, 1490 and 0, respectively. Second, the top-five high-potential THPs consisted of CFWPNRC (684), QWCSRRWCT (657), WTCRASWCS (632), SGWCYRC (631) and RWCREKSCW (631) that correspondingly had THP scores larger than 630. Third, note that almost all top 20 high-potential THPs would consist of at least two Cys residue with the exception of two peptides (i.e., WRPCES and WREWFL). Interesting, the top 20 non-THPs did not contain Cys residue in their primary sequences. Thus, we suggest that Cys residue and disulfide bonds may be important for THPs.

### 3.5. Characterization of THPs Using SCMTHP-Derived Propensity Scores

It is well recognized that THPs are beneficial for cancer therapy [1]. Insights from previous studies revealed that THPs had a typical length between 3 and 15 residues. Coincidentally, this has been reported to specifically recognize and bind tumor cells or tumor vasculature such as RGD peptides (bind to α_v_ integrins) and NGR peptides (bind to a receptor aminopeptidase N) [35,36]. Until now, many studies have attempted to identify and analyze THPs in terms of their selection and specification to different types of cancers. Herein, we proposed SCMTHP that was able to not only make predictions but also estimate the propensity scores of 20 amino acids in their contribution to THPs along with interpretation of their biological significance [15,16,17]. Figure 2 shows the propensity scores of 20 amino acids to be THPs as obtained from SCMTHP using Main-TRN (Figure 2A) and Small-TRN (Figure 2B) datasets. As already mentioned above, the propensity scores of 20 amino acids were obtained from Optimized-APS particularly from the 10th and 3rd experiments as evaluated on Main-TRN and Small-TRN, respectively. Note that amino acids exhibiting the highest propensity scores are also deemed to be the most important for tumor homing activity of peptides. In addition, Table 4 summarizes propensity scores of 20 amino acids to be THPs with corresponding amino acid compositions (%) of THP and non-THPs using the Main-TRN dataset. 

Several observations can be made from Table 4 as follows. (i) The five top-ranking amino acids having highest propensities for THPs included Cys, Trp, Arg, Pro and Phe with corresponding scores of 1000, 981, 598, 587 and 424, respectively, while five top-ranking amino acids with the lowest propensities for THPs were Ile, Lys, Val, Glu and Asp with corresponding scores of 0, 45, 48, 67 and 103, respectively. (ii) Cys, Trp, Arg and Pro with corresponding scores of 8.552, 2.371, 3.885 and 1.891, respectively, were the four top-ranking amino acids that correspondingly had the highest percentage difference of the composition. Meanwhile Ile, Lys, Val, Glu and Asp with corresponding scores of −3.066, −2.540, −2.514, −2.609 and −1.866, respectively, were the five top-ranking amino acids correspondingly having the lowest percentage difference of the composition. (iii) All of the four top-ranking amino acids having the highest and lowest propensities were significantly different with *p* < 0.01; they also had the largest correlation coefficient values between the propensity scores of 20 amino acids (PS-THP) and difference scores with values exceeding 0.8.

The aforementioned observation also confirmed the robustness of the SCMTHP-derived propensity scores of 20 amino acids for discriminating THPs from non-THPs. Such a result is consistent with computational analysis reported by several previous studies [13,14]. For example, Sharma et al. [13] reported that Cys, Arg, Gly, Trp, Pro, Leu and Ser are more abundant in THPs. Meanwhile, Shoombuatong et al. [14] showed that the three top-ranking informative amino acids were Cys, Trp and Arg, with corresponding mean decrease of the Gini index (MDGI) values of 139.48, 46.56 and 45.40, respectively. In the case of informative dipeptides, RC, GR, CR and CG were considered to be amongst the top four informative dipeptides [14]. Note that Cys might be beneficial for the tumor-homing activity of peptides. In 1997, Pasqualini et al. [37] showed that cyclic peptides having two disulfide bonds, such as the peptide sequence of CDCRGDCFC, could effectively bind to different integrins.

As shown in Table 2, the peptide sequence of CDCRGDCFC was found to be amongst the top 20 high-potential THPs that had a corresponding THP score of 598. The bicyclic CDCRGDCFC (RGD-4C) peptide is a ligand of integrins that can selectively bind αvβ3 and αvβ5 integrins, which are highly overexpressed on invading tumor endothelial cells and tumor vasculature [38]. Bicyclic forms of the peptide RGD-4C afforded less affinity for αvβ3 integrin and significantly less water solubility than the cyclic-(N-Me-VRGDf) (Cilengitide), which is a similar target of αvβ3 integrin [39]. Colombo et al. [40] compared the anticancer activity between cyclic (CNGRC-TNF) and linear (GNGRG-TNF) peptides containing the Asn-Gly-Arg (NGR) motif. Their results showed that the disulfide-bridge of the cyclic peptide afforded > 10 fold higher anti-tumor activity than that of the linear peptide. In addition, this group explored the dynamic behavior and conformational characteristics of NGR peptides with or without cyclic constraints by performing molecular dynamic (MD) simulations of two CNGRC peptides with and without disulfide bridges. Their analysis revealed that the disulfide bridge formation played a crucial role in the stabilization of the CNGRC peptide and enhancing the tumor targeting efficiency. Moreover, the insertion of a free Cys residue in investigated peptides could extend their half-life and binding affinities in tumors as reported by Pang et al. [41]. Particularly, they added a free Cys residue in the cyclic internalizing RGD (iRGD) tumor-targeting peptide (CRGDK/RGPD/EC), which led to longer half-life and more accumulation in tumors.

### 3.6. Characterization of THPs Using Informative PCPs

Several studies had reported that molecular weight [17], side chain [42,43], solubility [17], side chain [42,43] and beta-sheet propensity [44] were important factors for providing better understanding on functional mechanisms of proteins and peptides [16,21,24]. To be specific, from among several of these biochemical and biophysical properties, pI, hydrophobicity, side bulk, hydrophobicity, hydrophilicity and molecular weight have been reported to affect biological activities of peptides [13,45,46,47]. In this section, SCMTHP was applied for determining informative features from amongst the entire set of 531 PCPs in order to elucidate the relationship between THP scores and biochemical and biophysical properties. The set of 20 top-ranking informative PCPs having the largest R values are provided in Supplementary Table S11. Moreover, it could be noticed that the five top-ranking PCPs having the largest R values consisted of MCMT640101, ZASB820101, RACS820104, GARJ730101 and WIMW960101 with corresponding R values of 0.635, 0.623, 0.557, 0.512 and 0.507, respectively.

From among the five top-ranking PCPs with the largest R values, it was observed that the MCMT640101 property, described as the “Refractivity” [48], had the highest positive R value of 0.635. This can be attributed to two important factors (i.e., amino acid compositions and the refraction values of the amino acid residues) that affects the refractive indices. The high positive R value demonstrated that the refractivity property might be important for the functional mechanisms of THPs. McMeekin et al. described that the molar refraction property of amino acids can be measured by their aqueous solutions and via the Lorenz-Lorentz’s equation [48]. Their analysis showed that the refractive index is a unique characteristic of a protein that depends on the extinction coefficient (imaginary index) [49,50]. As can be observed from Table 5, the ranks of propensity scores (THP, refractivity, extinction coefficient) for Cys, Trp, Arg and Phe are (1, 2, 6), (2, 1, 1), (3, 5, 9) and (5, 4, 3), respectively. Furthermore, Kuipers and Gruppen reported that Trp exhibited the highest molar extinction coefficient of 29,050 while the second and third amino acids having the highest molar extinction coefficients were Tyr and Phe with respective extinction coefficients of 5375 and 5200, respectively. Note that the extinction coefficients of THPs might be higher than that of non-THPs. From Table 2 and Table 3, the mean, maximum and minimum scores extinction coefficients for the set of 20 top-ranked high-potential THPs are 6117.25, 12,615 and 125, respectively, while the mean, maximum and minimum extinction coefficients of the set of top 20 non-THPs are 149, 1490 and 0, respectively. In addition, note that the extinction coefficients for almost all of the 20 top-ranked high-potential THPs exceeded 149 with the exception of four peptides (i.e., CPRGSRC, CPHSKPCLC, CSRPRRSEC and CSRPRRSVC). In the same way, 18 out of 20 from the set of top 20 non-THPs exhibited extinction coefficients of 0. We also employed Student’s *t*-test to compare extinction coefficients of THPs and non-THPs on the Main-TRN dataset. It was found that the extinction coefficient was significant for the differentiation of THPs from non-THPs at the level of *p* < 0.001. These results indicated that the extinction coefficients of 20 amino acids were one of the important biochemical and biophysical properties governing THPs. As can be seen from Table 5, it can be noticed that aromatic amino acids (i.e., Phe, Tyr and His) presented π electron that can absorb UV light. It could be stated that peptides having high refractivity may have an accumulation of peptides in tumor cells and their environment. Moreover, the isoelectric point obtained from peptides with the highest THP score was mild acid/base, whereas the remaining was strong acid/base. However, peptides with the highest THP score also contained mostly neutral and positive net charge but the remaining contained negative net charge. From the above mentioned results, it can be deduced that cationic THPs may behave as cell penetrating and cytolytic peptides [51,52].

## 4. Conclusions

This study introduces SCMTHP as a novel, simple and interpretable scoring card (SCM)-based approach for in silico identification and characterization of THPs. The major contribution of the SCMTHP approach is the use of weighted-sum classifier as well as the new and improved propensity scores of 20 amino acids as THPs. Particularly, these propensity scores of 20 amino acids were used for identifying informative physicochemical properties that provided insights on characteristics of THPs. We have shown that SCMTHP could outperform almost all conventional ML-based predictors and state-of-the-art methods in terms of accuracy, conceptual simplicity and high interpretability in extensive comparative experiments on the two benchmark datasets. Furthermore, analysis revealed the significance of Cys residue in stabilization as well as a preference for high extinction coefficients. Finally, we have constructed a user-friendly online web server (http://pmlabstack.pythonanywhere.com/SCMTHP) (accessed on 27 December 2021) to facilitate online high-throughput THP identification. The SCMTHP predictor and SCMTHP-derived propensity scores of 20 amino acids are expected to be useful tools for facilitating THP identification and for improving our understanding of their functional mechanisms.

## Figures and Tables

**Table 1:** Performance comparison of SCMTHP with the existing method as evaluated by 10-fold cross-validation and independent tests.

Dataset	Cross-Validation	Method	ACC	Sn	Sp	MCC	AUC
Main	10-fold CV	THPred	0.857	0.877	0.837	0.716	0.929
		SCMTHP	0.820	0.819	0.820	0.641	0.869
	Independent test	THPred	0.846	0.792	0.900	0.696	0.939
		SCMTHP	0.827	0.869	0.785	0.656	0.869
Small	10-fold CV	THPred	0.824	0.781	0.867	0.653	0.893
		SCMTHP	0.808	0.723	0.893	0.628	0.853
	Independent test	THPred	0.798	0.862	0.734	0.601	0.885
		SCMTHP	0.798	0.766	0.830	0.597	0.853

**Table 2:** Top 20 peptides having the highest S(P) values along with their important physicochemical properties.

#	Sequence	THP Score	Length	Molecular Weight	Extinction Coefficient (M^−1^·cm^−1^)	pI	Net Charge	Hydrophobicity (Kcal·mol^−1^)
1	CFWPNRC	684	7	925.17	5625	8.30	1	6.86
2	QWCSRRWCT	657	9	1225.52	11125	8.93	2	8.78
3	WTCRASWCS	632	9	1099.35	11125	8.25	1	7.16
4	SGWCYRC	631	7	874.08	7115	8.21	1	8.48
5	RWCREKSCW	631	9	1253.57	11125	8.85	2	14.19
6	CSDWQHPWC	627	9	1161.39	11125	4.97	−1	11.02
7	CPRGSRC	621	7	777.99	125	9.66	2	13.23
8	CWRKFYC	617	7	1005.3	7115	9.24	2	7.96
9	CSDSWHYWC	615	9	1186.39	12615	4.97	−1	9.86
10	WRPCES	607	6	776.93	5500	6.16	0	11.83
11	CWLCNGRCGR	606	10	1167.52	5625	8.60	2	11.27
12	RHCFSQWCS	600	9	1153.41	5625	8.19	1	9.89
13	CDCRGDCFC	598	9	1021.26	250	3.91	−1	16.35
14	CPHSKPCLC	598	9	987.33	125	8.01	1	12.46
15	CWGCNGRCRM	595	10	1185.55	5625	8.60	2	11.85
16	CSRPRRSEC	585	9	1093.34	125	9.65	2	17.98
17	CSRPRRSVC	583	9	1063.36	125	11.33	3	13.89
18	CVLCNGRCWS	576	10	1140.49	5625	8.00	1	8.31
19	CRGDGWC	571	7	795.97	5625	5.94	0	13.52
20	WREWFL	571	6	936.16	11000	6.70	0	6.20
		610.25	8	1041.50	6117.25	7.82	1.00	11.05

**Table 3:** Top 20 peptides having the lowest S(P) values along with their important physicochemical properties.

#	Sequence	THP Score	Length	Molecular Weight	Extinction Coefficient (M^−1^·cm^−1^)	pI	Net Charge	Hydrophobicity (Kcal·mol^−1^)
1	IKIQD	69	5	615.80	0	6.72	0	12.87
2	KKEKDIMKKTI	74	11	1361.87	0	10.39	3	26.51
3	INGKVT	99	6	630.83	0	10.15	1	11.37
4	VKNNVEVN	105	8	915.13	0	6.81	0	15.50
5	IGIGAG	105	6	486.66	0	5.60	0	9.61
6	AVKKAYDIAIQ	108	11	1219.60	1490	9.73	1	16.00
7	DVGTTE	113	6	620.69	0	2.87	−2	16.36
8	IGDAT	114	5	475.56	0	3.00	−1	12.32
9	VAIDM	115	5	547.73	0	3.02	−1	9.79
10	DVKGVFVNI	119	9	990.30	0	6.77	0	12.13
11	DLAVVEVDQVMVVD	119	14	1530.96	0	2.63	−4	19.04
12	TDIDDKIINRAI	121	12	1386.74	0	4.21	−1	20.55
13	GDVVANT	123	7	674.80	0	3.00	−1	13.37
14	IDKQLE	131	6	744.93	0	7.00	−1	16.37
15	FGKKKKYKD	131	9	1141.50	1490	10.49	4	24.27
16	KENILNE	135	7	859.05	0	4.08	−1	17.29
17	HEAVGI	136	6	624.78	0	5.06	−1	13.93
18	HKNKGKKN	139	8	953.23	0	11.03	4	24.28
19	ENAKAAVAEMKDGDVVLLE	139	19	2002.54	0	3.84	−3	31.12
20	ITDMAA	140	6	620.79	0	3.13	−1	11.00
		116.75	8	920.17	149.00	5.98	−0.20	16.68

**Table 4:** Propensity scores of 20 amino acids to be THPs (PS-THP) along with amino acid compositions (%) of THPs and non-THPs based on the Main-TRN dataset.

Amino Acid	PS-THP	THP (%)	non-THP (%)	Difference	*p*-Value
C-Cys	1000(1)	9.635	1.082	8.552(1)	<0.01 *
W-Trp	981(2)	3.459	1.088	2.371(3)	<0.01 *
R-Arg	598(3)	8.947	5.062	3.885(2)	<0.01 *
P-Pro	587(4)	6.831	4.940	1.891(4)	<0.01 *
F-Phe	424(5)	3.018	3.846	−0.828(13)	0.017
S-Ser	407(6)	8.525	6.860	1.666(5)	<0.01 *
H-His	382(7)	3.084	2.699	0.385(6)	0.287
L-Leu	374(8)	8.157	9.394	−1.237(14)	0.020
Y-Tyr	273(9)	3.023	2.912	0.111(8)	0.741
M-Met	266(10)	2.629	2.604	0.025(9)	0.940
Q-Gln	198(11)	3.284	4.052	−0.769(11)	0.046
N-Asn	195(12)	3.365	4.169	−0.804(12)	0.033
A-Ala	160(13)	5.717	8.099	−2.382(16)	<0.01 *
G-Gly	157(14)	7.552	7.203	0.349(7)	0.516
T-Thr	150(15)	4.744	5.364	−0.620(10)	0.186
D-Asp	103(16)	3.798	5.664	−1.866(15)	<0.01 *
E-Glu	67(17)	3.544	6.153	−2.609(19)	<0.01 *
V-Val	48(18)	4.392	6.906	−2.514(17)	<0.01 *
K-Lys	45(19)	3.469	6.008	−2.540(18)	<0.01 *
I-Ile	0(20)	2.828	5.894	−3.066(20)	<0.01 *
R	1.000	0.462	−0.589	0.876	-

* Statistically significant at the level of *p*-value < 0.01.

**Table 5:** Summary of two important physicochemical properties (PCPs) as derived from SCMTHP.

Amino Acid	PS-THP	MCMT640101 ^a^	Molar Extinction Coefficients $(\varepsilon \;(M^{-1}\;{\mathrm{cm}}^{-1})){\;}^{b}$
C-Cys	1000(1)	35.77(2)	225(6)
W-Trp	981(2)	42.53(1)	29,050(1)
R-Arg	598(3)	26.66(5)	102(9)
P-Pro	587(4)	10.93(17)	30(19)
F-Phe	424(5)	29.4(4)	5200(3)
S-Ser	407(6)	6.35(18)	34(17)
H-His	382(7)	21.81(6)	5125(4)
L-Leu	374(8)	18.78(10)	45(13)
Y-Tyr	273(9)	31.53(3)	5375(2)
M-Met	266(10)	21.64(7)	980(5)
Q-Gln	198(11)	17.56(11)	142(7)
N-Asn	195(12)	13.28(14)	136(8)
A-Ala	160(13)	4.34(19)	32(18)
G-Gly	157(14)	0(20)	21(20)
T-Thr	150(15)	11.01(16)	41(16)
D-Asp	103(16)	12(15)	58(11)
E-Glu	67(17)	17.26(12)	78(10)
V-Val	48(18)	13.92(13)	43(14)
K-Lys	45(19)	21.29(8)	41(15)
I-Ile	0(20)	19.06(9)	45(12)
R	1.000	0.635	0.556

^a^ MCMT640101 = Refractivity (McMeekin et al., 1964) [18], Cited by Jones (1975) [18]. ^b^ ε (M^−1^ cm^−1^) ^c^ = Molar extinction coefficients (ε) of free amino acids (M^−1^ cm^−1^) at 214 nm in 20% (*v*/*v*) acetonitrile and 0.1% (*v*/*v*) formic acid derived from the work of [50].

## Data Availability

All the data are available at http://pmlabstack.pythonanywhere.com/SCMTHP (accessed on 27 December 2021).

## Supplementary Materials

The following supporting information can be downloaded at: https://www.mdpi.com/article/10.3390/pharmaceutics14010122/s1, Figure S1: Performance evaluations of SCMTHP and other ML-based classifiers in terms of ACC and MCC as evaluated by 10-fold cross-validation (A,B) and independent (C,D) tests on the Small-TRN and Small-IND datasets, respectively; Table S1: Hyperparameter search details for seven popular ML algorithms; Table S2: Cross-validation of ten SCM models trained with ten different sets of propensity scores of amino acids on the Main-TRN dataset; Table S3: Independent test results of ten SCM models trained with ten different sets of propensity scores of amino acids on the Main-IND dataset, respectively; Table S4: Cross-validation of ten SCM models trained with ten different sets of propensity scores of amino acids on the Small-TRN dataset; Table S5: Independent test results of ten SCM models trained with ten different sets of propensity scores of amino acids on the Small-IND dataset, respectively; Table S6: Cross-validation results of seven different ML classifiers with five different feature encodings on Main-TRN dataset; Table S7: Independent test results of seven different ML classifiers with five different feature encodings on Main-IND dataset; Table S8: Cross-validation results of seven different ML classifiers with five different feature encodings on Small-TRN dataset; Table S9: Independent test results of seven different ML classifiers with five different feature encodings on Small-IND dataset; Table S10: Cross-validation and independent test results of SCM-based classifiers by using Initial-APS and Optimized-APS as evaluated on the Main and Small datasets; Table S11: The twenty top-ranked informative physicohemical properties having the highest pearson correlation (R) with the propensity scores of amino acids on Main-TRN dataset.
