# Supplementary material for: SCMTHP: A New Approach for Identifying and Characterizing of Tumor-Homing Peptides Using Estimated Propensity Scores of Amino Acids
Source: Pharmaceutics. 2022 Jan 4;14(1):122. doi: 10.3390/pharmaceutics14010122 (PMC8779003; doi:10.3390/pharmaceutics14010122)
Supplement: Supplementary file 1 [file pharmaceutics-14-00122-s001.zip › pharmaceutics-1484097-supplementary.pdf]

pharmaceutics

Supplementary Materials: SCMTHP: A New Approach for Identifying and Characterizing of Tumor-Homing Peptides Using Estimated Propensity Scores of Amino Acids

Phasit Charoenkwan, Wararat Chiangjong, Chanin Nantasenamat, Mohammad Ali Moni, Pietro Lio', Balachandran Manavalan and Watshara Shoombuatong

GA algorithm of for optimizing propensity scores of 20 amino acids

The GA algorithm of the SCM for optimizing Initial-APS consists of the following steps:
Step 1: (Initialization) Generate randomly 40 sets of APS including the Initial-APS.
Step 2: (Evaluation) Compute fitness values from the fitness function (Eq.1) for all sets of APS to determine the best APS in the population.
Step 3: (Crossover) Perform a 20-point crossover between the best APS and each other APS.
Step 4: (Mutation) Randomly mutate individuals (except the best APS) with a mutation probability Pm (=0.01), using a real-valued mutation operator.
Step 5: (Termination) Stop the GA algorithm if the termination condition is reached, otherwise, go to the Step 2. In this study, 20 generations are used as the stop condition.
More details on the optimization dipeptides propensity scores using GA algorithm can be found in previous studies [1-4].

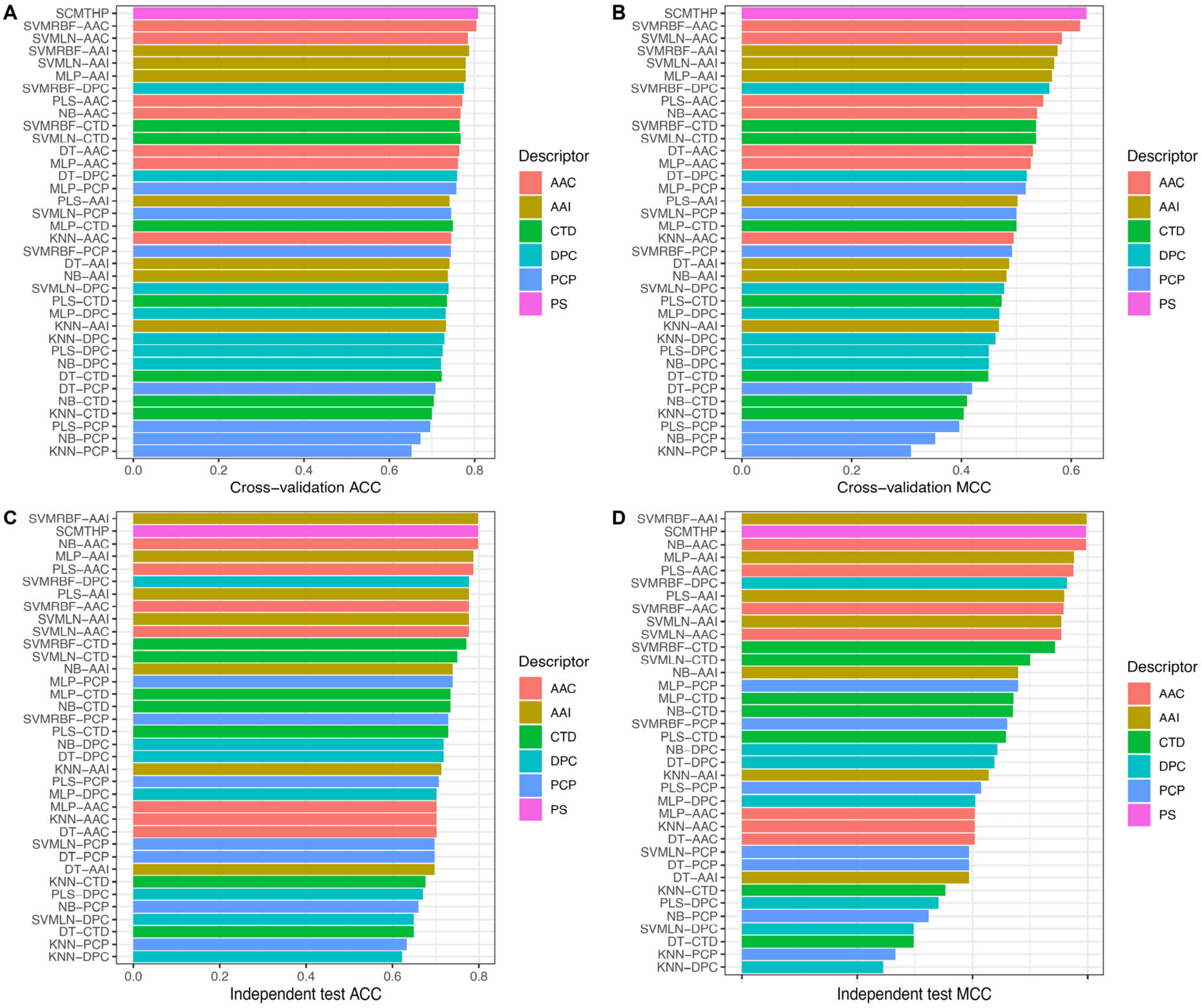

Figure S1. Performance evaluations of SCMTHP and other ML-based classifiers in terms of ACC and MCC as evaluated by 10-fold cross-validation (**A**,**B**) and independent (**C**,**D**) tests on the Small-TRN and Small-IND datasets, respectively.

Table S1. Hyperparameter search details for seven popular ML algorithms.

Method	Parameters	Range of parameters
DT	max_depth	Default
KNN	number of neighbours	Default
MLP	hidden_layer_sizes	[50, 100, 300, 500]
NB	sample_weight	Default parameter
PLS	#Components	Default parameter
SVMLN	Cost	[2^0–2^5] in $\log_2$ steps
SVMRBF	Cost	[2^0–2^5] in $\log_2$ steps

Columns 2 and 3 represent the parameter name used in the Scikit-learn library and the range of parameter used to develop the model, respectively.

Table S2. Cross-validation of ten SCM models trained with ten different sets of propensity scores of amino acids on the Main-TRN dataset.

#Exp	Cutoff	ACC	Sn	Sp	MCC	AUC
1	251	0.808	0.800	0.816	0.619	0.868
2	330	0.814	0.802	0.825	0.629	0.866
3	237	0.808	0.798	0.818	0.619	0.865
4	238	0.810	0.829	0.791	0.623	0.870

5	318	0.803	0.756	0.850	0.611	0.851
6	315	0.809	0.810	0.808	0.619	0.858
7	253	0.815	0.818	0.812	0.631	0.868
8	243	0.809	0.831	0.787	0.621	0.868
9	331	0.808	0.781	0.835	0.618	0.860
**10**	**301**	**0.820**	**0.819**	**0.820**	**0.641**	**0.869**
Mean		0.810	0.805	0.816	0.623	0.864
STD.		0.005	0.023	0.019	0.009	0.006

The 10th experiment having the highest cross-validation ACC is used for further analysis in this study.

Table S3. Independent test results of ten SCM models trained with ten different sets of propensity scores of amino acids on the Main-IND dataset, respectively.

#Exp	Cutoff	ACC	Sn	Sp	MCC	AUC
1	251	0.792	0.838	0.746	0.587	0.868
2	330	0.777	0.831	0.723	0.557	0.866
3	237	0.773	0.823	0.723	0.549	0.865
4	238	0.785	0.846	0.723	0.574	0.870
5	318	0.819	0.854	0.785	0.640	0.851
6	315	0.800	0.838	0.762	0.602	0.858
7	253	0.796	0.846	0.746	0.595	0.868
8	243	0.785	0.862	0.708	0.576	0.868
9	331	0.796	0.815	0.777	0.593	0.860
**10**	**301**	**0.827**	**0.869**	**0.785**	**0.656**	**0.869**
Mean		0.795	0.842	0.748	0.593	0.864
STD.		0.017	0.017	0.028	0.034	0.006

The 10th experiment having the highest cross-validation ACC is used for further analysis in this study.

Table S4. Cross-validation of ten SCM models trained with ten different sets of propensity scores of amino acids on the Small-TRN dataset.

#Exp	Cutoff	ACC	Sn	Sp	MCC	AUC
1	326	0.804	0.760	0.848	0.611	0.846
2	365	0.799	0.789	0.808	0.599	0.837
**3**	**333**	**0.808**	**0.723**	**0.893**	**0.628**	**0.853**
4	299	0.805	0.744	0.867	0.619	0.855
5	268	0.804	0.747	0.861	0.614	0.862
6	313	0.805	0.744	0.867	0.617	0.852
7	321	0.807	0.755	0.859	0.618	0.846
8	288	0.799	0.776	0.821	0.600	0.846
9	304	0.803	0.792	0.813	0.607	0.857
10	279	0.799	0.707	0.891	0.612	0.859
Mean		0.803	0.754	0.853	0.612	0.851
STD.		0.003	0.027	0.030	0.009	0.007

The 3rd experiment having the highest cross-validation ACC is used for further analysis in this study.

Table S5. Independent test results of ten SCM models trained with ten different sets of propensity scores of amino acids on the Small-IND dataset, respectively.

#Exp	Cutoff	ACC	Sn	Sp	MCC	AUC
1	326	0.777	0.809	0.745	0.554	0.846
2	365	0.729	0.766	0.691	0.459	0.837

**3**	**333**	**0.798**	**0.766**	**0.830**	**0.597**	**0.853**
4	299	0.761	0.766	0.755	0.521	0.855
5	268	0.777	0.809	0.745	0.554	0.862
6	313	0.787	0.798	0.777	0.575	0.852
7	321	0.718	0.745	0.691	0.437	0.846
8	288	0.745	0.819	0.670	0.495	0.846
9	304	0.766	0.840	0.691	0.538	0.857
10	279	0.787	0.766	0.809	0.575	0.859
Mean		0.764	0.788	0.740	0.530	0.851
STD.		0.026	0.031	0.054	0.053	0.007

The 3rd experiment having the highest cross-validation ACC is used for further analysis in this study.

Table S6. Cross-validation results of seven different ML classifiers with five different feature encodings on Main-TRN dataset.

Descriptor	Method	Parameter	ACC	Sn	Sp	MCC	AUC
AAC	DT	N/A	0.775	0.779	0.772	0.553	0.775
	KNN	N/A	0.786	0.743	0.829	0.579	0.786
	MLP	100	0.794	0.800	0.787	0.590	0.870
	NB	N/A	0.775	0.710	0.841	0.558	0.854
	PLS	N/A	0.791	0.747	0.835	0.585	0.860
	SVMLN	32	0.799	0.766	0.833	0.602	0.866
	SVMRBF	4	0.833	0.835	0.831	0.669	0.902
AAI	DT	N/A	0.738	0.743	0.733	0.478	0.738
	KNN	N/A	0.772	0.741	0.802	0.545	0.772
	MLP	100	0.805	0.768	0.842	0.618	0.882
	NB	N/A	0.743	0.683	0.802	0.491	0.805
	PLS	N/A	0.771	0.693	0.848	0.552	0.845
	SVMLN	4	0.800	0.768	0.833	0.604	0.866
	SVMRBF	2	0.819	0.796	0.841	0.639	0.894
CTD	DT	N/A	0.731	0.729	0.733	0.464	0.731
	KNN	N/A	0.722	0.687	0.756	0.447	0.722
	MLP	50	0.796	0.800	0.791	0.594	0.867
	NB	N/A	0.707	0.683	0.731	0.417	0.776
	PLS	N/A	0.763	0.747	0.779	0.527	0.832
	SVMLN	1	0.815	0.802	0.827	0.631	0.883
	SVMRBF	4	0.798	0.768	0.827	0.598	0.880
DPC	DT	N/A	0.729	0.733	0.726	0.463	0.729
	KNN	N/A	0.725	0.536	0.914	0.485	0.725
	MLP	100	0.776	0.728	0.823	0.556	0.840
	NB	N/A	0.764	0.708	0.819	0.533	0.788
	PLS	N/A	0.762	0.732	0.793	0.527	0.820
	SVMLN	1	0.753	0.724	0.783	0.510	0.822
	SVMRBF	8	0.807	0.846	0.768	0.619	0.898
PCP	DT	N/A	0.717	0.758	0.676	0.438	0.717
	KNN	N/A	0.731	0.728	0.735	0.464	0.731
	MLP	100	0.771	0.772	0.770	0.545	0.847
	NB	N/A	0.690	0.643	0.737	0.382	0.755
	PLS	N/A	0.701	0.674	0.727	0.402	0.772
	SVMLN	2	0.734	0.710	0.758	0.471	0.796
	SVMRBF	8	0.762	0.749	0.776	0.527	0.832

Table S7. Independent test results of seven different ML classifiers with five different feature encodings on Main-IND dataset.

Descriptor	Method	Parameter	ACC	Sn	Sp	MCC	AUC
AAC	DT	N/A	0.812	0.823	0.800	0.623	0.812
	KNN	N/A	0.785	0.831	0.738	0.572	0.785
	MLP	100	0.800	0.854	0.746	0.604	0.879
	NB	N/A	0.804	0.823	0.785	0.608	0.894
	PLS	N/A	0.800	0.823	0.777	0.601	0.875
	SVMLN	32	0.808	0.838	0.777	0.617	0.888
	SVMRBF	4	0.804	0.838	0.769	0.609	0.894
AAI	DT	N/A	0.750	0.815	0.685	0.504	0.750
	KNN	N/A	0.796	0.754	0.838	0.594	0.796
	MLP	100	0.808	0.792	0.823	0.616	0.888
	NB	N/A	0.765	0.800	0.731	0.532	0.827
	PLS	N/A	0.785	0.808	0.762	0.570	0.864
	SVMLN	4	0.804	0.838	0.769	0.609	0.888
	SVMRBF	2	0.827	0.869	0.785	0.656	0.903
CTD	DT	N/A	0.762	0.769	0.754	0.523	0.762
	KNN	N/A	0.781	0.785	0.777	0.562	0.781
	MLP	50	0.808	0.915	0.700	0.630	0.923
	NB	N/A	0.738	0.762	0.715	0.477	0.787
	PLS	N/A	0.777	0.815	0.738	0.555	0.855
	SVMLN	1	0.812	0.915	0.708	0.637	0.887
	SVMRBF	4	0.846	0.862	0.831	0.693	0.920
DPC	DT	N/A	0.742	0.792	0.692	0.487	0.742
	KNN	N/A	0.696	0.508	0.885	0.424	0.696
	MLP	100	0.758	0.762	0.754	0.515	0.834
	NB	N/A	0.785	0.800	0.769	0.570	0.816
	PLS	N/A	0.782	0.791	0.773	0.564	0.840
	SVMLN	1	0.746	0.769	0.723	0.493	0.803
	SVMRBF	8	0.777	0.838	0.715	0.558	0.863
PCP	DT	N/A	0.727	0.815	0.638	0.461	0.730
	KNN	N/A	0.727	0.746	0.708	0.454	0.727
	MLP	100	0.785	0.823	0.746	0.571	0.901
	NB	N/A	0.727	0.746	0.708	0.454	0.792
	PLS	N/A	0.735	0.762	0.708	0.470	0.816
	SVMLN	2	0.765	0.800	0.731	0.532	0.863
	SVMRBF	8	0.788	0.862	0.715	0.583	0.877

Table S8. Cross-validation results of seven different ML classifiers with five different feature encodings on Small-TRN dataset.

Descriptor	Method	Parameter	ACC	Sn	Sp	MCC	AUC
AAC	DT	N/A	0.764	0.771	0.757	0.530	0.764
	KNN	N/A	0.745	0.771	0.720	0.495	0.745
	MLP	50	0.761	0.787	0.736	0.526	0.845
	NB	N/A	0.767	0.731	0.803	0.538	0.836
	PLS	N/A	0.771	0.701	0.840	0.549	0.850
	SVMLN	1	0.784	0.680	0.888	0.583	0.846
	SVMRBF	1	0.804	0.731	0.877	0.616	0.872
AAI	DT	N/A	0.741	0.766	0.717	0.487	0.741
	KNN	N/A	0.733	0.738	0.728	0.468	0.733

	MLP	100	0.779	0.738	0.818	0.565	0.864
	NB	N/A	0.737	0.677	0.797	0.482	0.805
	PLS	N/A	0.741	0.624	0.859	0.502	0.841
	SVMLN	1	0.779	0.693	0.864	0.569	0.848
	SVMRBF	8	0.787	0.781	0.792	0.575	0.856
CTD	DT	N/A	0.723	0.720	0.725	0.449	0.723
	KNN	N/A	0.700	0.739	0.661	0.404	0.700
	MLP	100	0.749	0.739	0.759	0.500	0.828
	NB	N/A	0.704	0.726	0.682	0.410	0.778
	PLS	N/A	0.735	0.689	0.781	0.473	0.799
	SVMLN	1	0.767	0.736	0.797	0.536	0.827
	SVMRBF	4	0.765	0.723	0.807	0.536	0.838
DPC	DT	N/A	0.759	0.755	0.763	0.519	0.759
	KNN	N/A	0.729	0.725	0.733	0.462	0.729
	MLP	50	0.732	0.726	0.739	0.469	0.800
	NB	N/A	0.721	0.640	0.802	0.450	0.737
	PLS	N/A	0.725	0.720	0.729	0.450	0.797
	SVMLN	1	0.739	0.733	0.744	0.478	0.802
	SVMRBF	1	0.775	0.683	0.866	0.560	0.848
PCP	DT	N/A	0.708	0.739	0.678	0.419	0.711
	KNN	N/A	0.652	0.689	0.616	0.308	0.652
	MLP	100	0.757	0.733	0.781	0.517	0.829
	NB	N/A	0.673	0.736	0.611	0.352	0.750
	PLS	N/A	0.696	0.673	0.720	0.396	0.779
	SVMLN	2	0.745	0.659	0.832	0.500	0.805
	SVMRBF	1	0.744	0.691	0.797	0.492	0.811

Table S9. Independent test results of seven different ML classifiers with five different feature encodings on Small-IND dataset.

Descriptor	Method	Parameter	ACC	Sn	Sp	MCC	AUC
AAC	DT	N/A	0.702	0.702	0.702	0.404	0.702
	KNN	N/A	0.702	0.713	0.691	0.404	0.702
	MLP	50	0.702	0.713	0.691	0.404	0.788
	NB	N/A	0.798	0.830	0.766	0.597	0.863
	PLS	N/A	0.787	0.777	0.798	0.575	0.856
	SVMLN	1	0.777	0.745	0.809	0.554	0.839
	SVMRBF	1	0.777	0.713	0.840	0.558	0.837
AAI	DT	N/A	0.697	0.681	0.713	0.394	0.697
	KNN	N/A	0.713	0.766	0.660	0.428	0.713
	MLP	100	0.787	0.755	0.819	0.576	0.865
	NB	N/A	0.739	0.745	0.734	0.479	0.814
	PLS	N/A	0.777	0.702	0.851	0.559	0.849
	SVMLN	1	0.777	0.745	0.809	0.554	0.840
	SVMRBF	8	0.798	0.755	0.840	0.598	0.845
CTD	DT	N/A	0.649	0.681	0.617	0.298	0.649
	KNN	N/A	0.676	0.734	0.617	0.353	0.676
	MLP	100	0.734	0.787	0.681	0.471	0.828
	NB	N/A	0.734	0.777	0.691	0.470	0.795
	PLS	N/A	0.729	0.713	0.745	0.458	0.829
	SVMLN	1	0.750	0.766	0.734	0.500	0.835
	SVMRBF	4	0.771	0.755	0.787	0.543	0.845

DPC	DT	N/A	0.718	0.766	0.670	0.438	0.718
	KNN	N/A	0.622	0.606	0.638	0.245	0.622
	MLP	50	0.702	0.681	0.723	0.405	0.747
	NB	N/A	0.718	0.628	0.809	0.443	0.720
	PLS	N/A	0.670	0.649	0.691	0.341	0.758
	SVMLN	1	0.649	0.660	0.638	0.298	0.697
	SVMRBF	1	0.777	0.681	0.872	0.564	0.825
PCP	DT	N/A	0.697	0.713	0.681	0.394	0.701
	KNN	N/A	0.633	0.649	0.617	0.266	0.633
	MLP	100	0.739	0.723	0.755	0.479	0.811
	NB	N/A	0.660	0.745	0.574	0.324	0.720
	PLS	N/A	0.707	0.702	0.713	0.415	0.738
	SVMLN	2	0.697	0.702	0.691	0.394	0.771
	SVMRBF	1	0.729	0.681	0.777	0.460	0.779

Table S10. Cross-validation and independent test results of SCM-based classifiers by using Initial-APS and Optimized-APS as evaluated on the Main and Small datasets.

Dataset	Cross-validation	Feature	ACC	Sn	Sp	MCC	AUC
Main	10-fold CV	Initial-APS	0.752	0.695	0.810	0.511	0.831
		Optimized-APS	0.820	0.819	0.820	0.641	0.869
	Independent test	Initial-APS	0.738	0.792	0.685	0.480	0.831
		Optimized-APS	0.827	0.869	0.785	0.656	0.869
Small	10-fold CV	Initial-APS	0.739	0.747	0.731	0.479	0.840
		Optimized-APS	0.808	0.723	0.893	0.628	0.853
	Independent test	Initial-APS	0.729	0.798	0.660	0.462	0.840
		Optimized-APS	0.798	0.766	0.830	0.597	0.853

Table S11. The twenty top-ranked informative physicohemical properties having the highest pearson correlation (R) with the propensity scores of amino acids on Main-TRN dataset.

Rank	AAindex	R	Description
1	MCMT640101	0.635	Refractivity (McMeekin et al., 1964), Cited by Jones (1975)
2	ZASB820101	0.623	Dependence of partition coefficient on ionic strength (Zaslavsky et al., 1982)
3	RACS820104	0.557	Average relative fractional occurrence in EL(i) (Rackovsky-Scheraga, 1982)
4	GARJ730101	0.512	Partition coefficient (Garel et al., 1973)
5	WIMW960101	0.507	Free energies of transfer of AcWl-X-LL peptides from bilayer interface to water (Wimley-White, 1996)
6	CHOP780215	0.492	Frequency of the 4th residue in turn (Chou-Fasman, 1978b)
7	MEEJ810101	0.476	Retention coefficient in NaClO4 (Meek-Rossetti, 1981)
8	CASG920101	0.465	Hydrophobicity scale from native protein structures (Casari-Sippl, 1992)
9	WOLS870103	0.457	Principal property value z3 (Wold et al., 1987)
10	ROBB760110	0.45	Information measure for middle turn (Robson-Suzuki, 1976)
11	WERD780101	0.445	Propensity to be buried inside (Wertz-Scheraga, 1978)
12	ZHOH040103	0.442	Buriability (Zhou-Zhou, 2004)
13	SUYM030101	0.44	Linker propensity index (Suyama-Ohara, 2003)

14	ZHOH040101	0.435	The stability scale from the knowledge-based atom-atom potential (Zhou-Zhou, 2004)
15	WILM950103	0.434	Hydrophobicity coefficient in RP-HPLC, C4 with 0.1%TFA/MeCN/H2O (Wilce et al. 1995)
16	QIAN880127	0.434	Weights for coil at the window position of -6 (Qian-Sejnowski, 1988)
17	BAEK050101	0.434	Linker index (Bae et al., 2005)
18	RICJ880107	0.432	Relative preference value at N4 (Richardson-Richardson, 1988)
19	ROBB760108	0.429	Information measure for turn (Robson-Suzuki, 1976)
20	LEVM760107	0.426	van der Waals parameter epsilon (Levitt, 1976)

References

1. P. Charoenkwan, W. Shoombuatong, H.-C. Lee, J. Chaijaruwanich, H.-L. Huang, and S.-Y. Ho, "SCMCRYS: predicting protein crystallization using an ensemble scoring card method with estimating propensity scores of P-collocated amino acid pairs," *PloS one*, vol. 8, no. 9, p. e72368, 2013.
2. H.-L. Huang *et al.*, "Prediction and analysis of protein solubility using a novel scoring card method with dipeptide composition," in *BMC bioinformatics*, 2012, vol. 13, no. 17, pp. 1-14: Springer.
3. P. Charoenkwan, W. Chiangjong, V. S. Lee, C. Nantasenamat, M. M. Hasan, and W. Shoombuatong, "Improved prediction and characterization of anticancer activities of peptides using a novel flexible scoring card method," *Scientific reports*, vol. 11, no. 1, pp. 1-13, 2021.
4. P. Charoenkwan, N. Schaduangrat, C. Nantasenamat, T. Piacham, and W. Shoombuatong, "iQSP: A Sequence-Based Tool for the Prediction and Analysis of Quorum Sensing Peptides Using Informative Physicochemical Properties," *International Journal of Molecular Sciences*, vol. 21, no. 1, p. 75, 2020.
